# Penile mass with a past: Tertiary syphilis presenting as a penile gumma

**DOI:** 10.1016/j.eucr.2025.103279

**Published:** 2025-11-17

**Authors:** Gabriel E. Martin, Sharon Wang, Nolan S. Maloney, Dan Xu, Herbert Ruckle, Martin R. Hofmann, Brian R. Hu

**Affiliations:** a Department of Urology, Loma Linda University Health, 11234 Anderson Street, Loma Linda, CA, 92354, USA; b San Bernardino County Department of Public Health, 451 E. Vanderbilt Way, San Bernardino, CA, 92408, USA; c Department of Pathology, Loma Linda University Health, 11234 Anderson Street, Loma Linda, CA, 92354, USA

**Keywords:** Tertiary syphilis, Penile gumma, *Treponema pallidum*, Seronegative syphilis, Penile mass, Penile biopsy, Immunohistochemistry

## Abstract

A 46-year-old man presented with a rapidly growing, verrucous penile mass with bilateral inguinal lymphadenopathy, clinically consistent with locally advanced penile carcinoma. He had no history of sexually transmitted infections and negative rapid plasma reagin (RPR) testing. Biopsy demonstrated no malignancy, but immunohistochemistry revealed *Treponema pallidum*. He was diagnosed with tertiary syphilis and penile gumma. He was treated with penicillin G, though the gumma showed minimal response and surgical extirpation was recommended. This case highlights the limitations of traditional RPR-first screening in syphilis and underscores the importance of a broad differential diagnosis when addressing penile masses.

## Highlights

• Tertiary syphilis presented as penile gumma mimicking malignancy.
• Penile mass biopsy IHC diagnosed the *Treponema pallidum* gumma.
• Serum testing with RPR was negative.
• Use of reverse algorithm testing with antibodies is better in late-stage syphilis.

## 1 Introduction

Syphilis, caused by the spirochete *Treponema pallidum*, is known as “the great pretender” because it mimics a wide variety of diseases across organ systems.^1^^,^^2^ Primary syphilis is classically a painless ulcer (chancre). When untreated, it progresses to secondary syphilis known for various disseminated dermatologic manifestations and systemic symptoms. In latent syphilis, the host immune response clears the infection from primary and secondary lesions and resolves clinical manifestations, but spirochetes remain systemically. Tertiary syphilis can present decades after initial infection, and includes central nervous system involvement, cardiovascular disease, or destructive lesions called syphilitic gumma.^3^^,^^4^

**Fig. 1:**
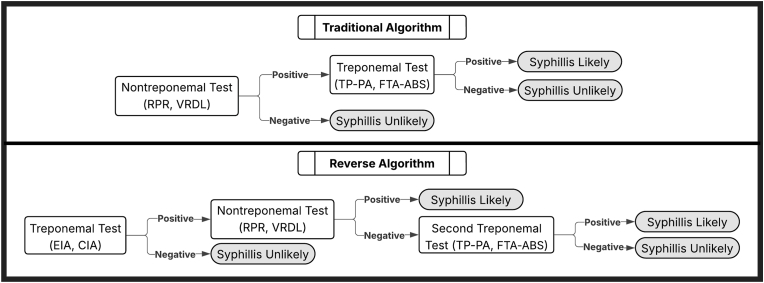
Traditional and reverse syphilis screening algorithms (adapted from CDC guidelines).^11^

Although syphilis can be effectively treated with penicillin, it remains a worldwide health concern and its incidence has been increasing in high income countries including the United States.^5^ Direct detection methods of syphilis include immunohistochemistry (IHC) and direct fluorescent antibody (DFA) staining, darkfield microscopy (DFM), and nucleic acid detection.^6^ More frequently used is serological testing, which includes treponemal tests looking for specific antibodies against *T*. *pallidum* (i.e. enzyme immunoassay (EIA), chemiluminescence immunoassays (CIA), *Treponema pallidum* particle agglutination (TP-PA) and fluorescent treponemal antibody absorption (FTA-ABS)) and non-treponemal tests looking for nonspecific antibodies created in response to damaged cells (i.e. rapid plasma reagin (RPR), venereal disease research laboratory (VDRL) test).^7^ There are two commonly accepted treatment algorithms, the traditional algorithm which screens with a nontreponemal test and confirms with a treponemal test and the reverse algorithm which screens with a treponemal test and confirms with a nontreponemal test (Fig. 1).^8^ Latent syphilis can sero-revert to a negative nontreponemal serology (i.e. RPR or VRDL) without evidence of disease, or retain positive nontreponemal serology without evidence of disease, or develop tertiary syphilis.^4^ Recent studies suggest that the reverse algorithm may be better at detecting syphilis, particularly important in late stage (latent and tertiary) syphilis who may have seronegative testing by the traditional algorithm.^8^, ^9^, ^10^, ^11^ Additionally, reverse algorithm may be preferred in areas with high prevalence of syphilis.^12^

*T. pallidum* has low surface antigenicity, allowing it to evade immune clearance and persist in host tissue.^5^ Syphilitic gummas represent a proliferative granulomatous process in response to persistent *T. pallidum,* and can occur in any tissue.^13^ Penile gummas represent one of the rarest presentations of tertiary syphilis, with fewer than twenty cases reported in the literature.^14^^,^^15^ We present a unique case of seronegative tertiary syphilis presenting as a penile gumma mimicking penile carcinoma. This highlights the challenge of the traditional algorithm in diagnosing tertiary syphilis and emphasizes the importance of considering an infectious etiology of a penile mass.

## 2 Case report

A 46-year-old uncircumcised man presented to the emergency department with a growing penile mass with associated penile pain, dysuria, and obstructive lower urinary tract symptoms. He reported that a small penile lesion, present “for as long as he could remember,” began rapidly enlarging for the five months prior to presentation. His medical conditions included diabetes, hypertension, and hyperlipidemia with no known history of sexually transmitted infections. He was married for over 15 years in a monogamous relationship, although he had several sexual partners prior to marriage.

**Fig. 2:**
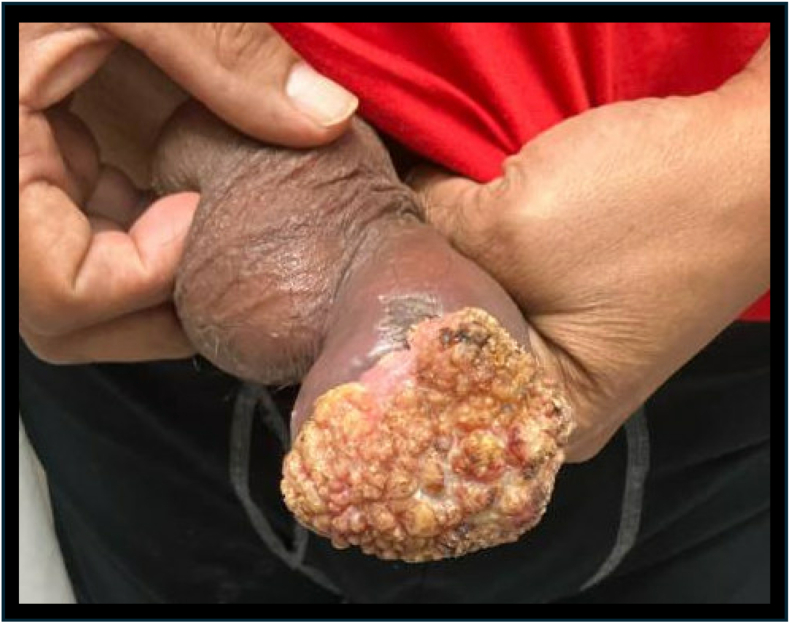
Penile mass at initial presentation showing large, exophytic, verrucous mass completely obscuring the urethral meatus.

His physical exam showed a large, exophytic, and verrucous mass encapsulating the glans penis and obscuring the urethral meatus with induration of the distal corpora cavernosa (Fig. 2). Bilateral, nontender inguinal lymphadenopathy was palpable. He had a mild leukocytosis (13.06 × 10^3^ WBC/μL) and computed tomography (CT) revealed bilateral inguinal and right external iliac lymphadenopathy suspicious for metastatic disease. CT chest was negative for evidence of malignancy.

**Fig. 3:**
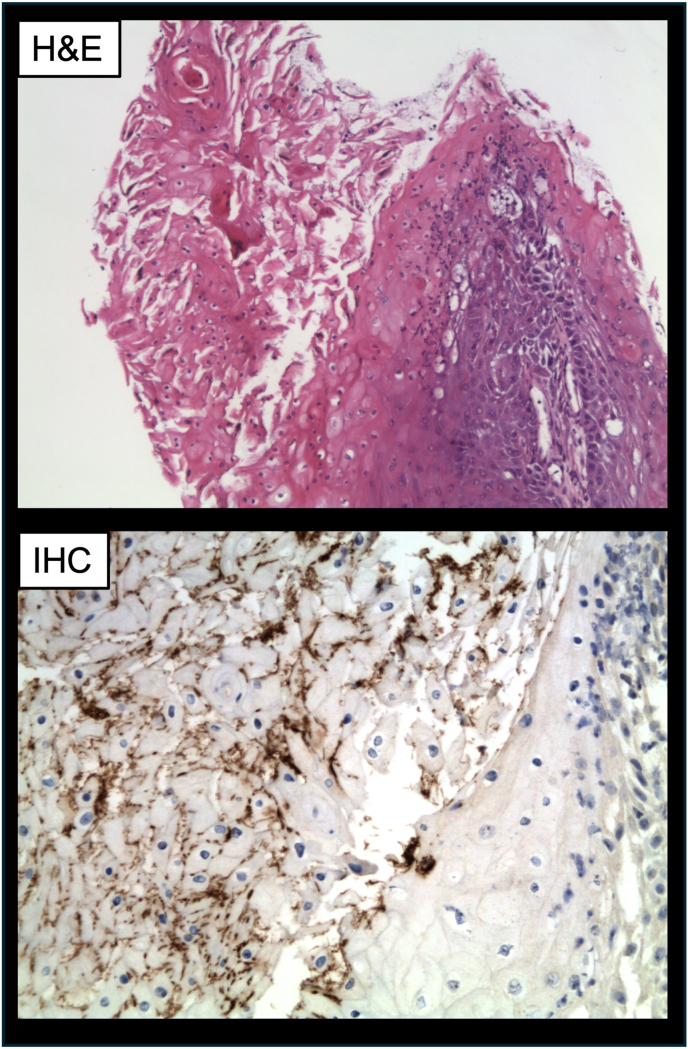
Microscopic evaluation of mass core biopsy with hematoxylin and eosin staining (H&E) and spirochete immunohistochemistry (IHC) staining with spirochetes stained dark brown.

Core biopsies were taken of the distal corpora and penile mass in the emergency department, and the patient was discharged home with close urology follow up. Initial pathology revealed reactive changes of the skin, extensive fibrosis, and patchy mixed acute and chronic inflammation with granulation tissue in all specimens. No evidence of malignancy was identified. Given the benign pathology, further spirochete-specific immunohistochemical staining was performed and was positive for organisms morphologically compatible with *T*. *pallidum* (Fig. 3).

**Fig. 4:**
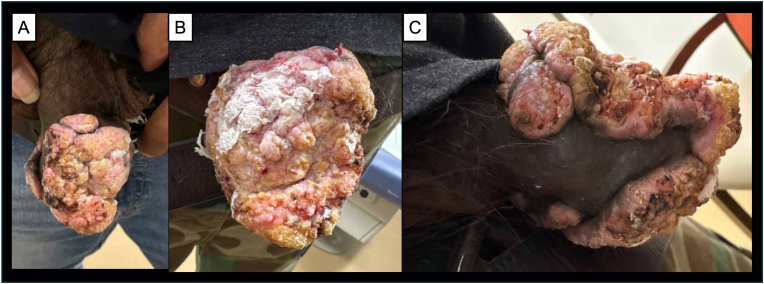
Penile mass prior to first dose of penicillin (Panel A) and penile mass 2 weeks later immediately following third dose of penicillin seen dorsally (Panel B) and ventrally (Panel C).

He was seen by infectious disease and was diagnosed with tertiary syphilis, and the penile mass was identified as a gumma. He had two nontreponemal tests (RPR) that were both nonreactive. Subsequent treponemal tests were ordered but not completed. He was treated with the standard three weekly injections of penicillin G with worsening of the gumma (Fig. 4 demonstrates the mass after each weekly treatment). The patient followed up in urology clinic and partial penectomy was recommended. The patient declined the surgery.

## 3 Discussion

In this case, a tertiary syphilis penile gumma was clinically and radiographically indistinguishable from penile carcinoma. A broad differential diagnosis, utilization of biopsy, and re-staining of the benign specimen for spirochetes led to the diagnosis. This represents one of fewer than twenty cases of reported penile gumma, with his seronegative presentation and treatment response failure being even more rare.^14^^,^^15^ With the recent resurgence of syphilis, increased awareness of its clinical presentations, methods of diagnosis, and treatment is warranted.^5^

This case of gummatous syphilis highlights:

1) The limitations of traditional RPR-first screening in late-stage syphilis. Although data on tertiary syphilis is sparse, review of international data shows that nontreponemal tests have poor sensitivity in late-stage syphilis compared to the treponemal tests employed in the reverse algorithm.^8^, ^9^, ^10^, ^11^ With cases of syphilis on the rise, clinicians should remain aware of the reverse algorithm and consider ordering treponemal serologic testing in suspected late-stage syphilis, or if concern for syphilis persists despite negative nontreponemal testing.
2) Treatment challenges in tertiary syphilis. In several prior case reports of penile and testicular gummas, appropriate treatment resulted in resolution of lesions.^16^, ^17^, ^18^ It is therefore important to attempt antibiotic treatment prior to extirpation in tertiary syphilis in an attempt to reduce morbidity. Our patient's presentation with a sizable mass with irreversible fibrotic changes stands out compared to prior case reports which have documented response to penicillin treatment.
3) The utility of biopsy preceding partial penectomy. The most recent European Association of Urology and American Society of Clinical Oncology collaborative guidelines on penile cancer recommend biopsy of the primary tumor when there is doubt about the exact nature of the lesion.^19^ As biopsy can be performed relatively easily and promptly, consideration should be made for this step prior to partial or total penectomy. While no guidelines exist for biopsy of suspected gummas, our experience is that adequate sampling should include core biopsy with sufficient depth to capture viable tissue. Gummas are often centrally necrotic, so sampling the periphery of the lesion may yield more viable tissue for histopathologic evaluation. In our case, core biopsies of both the mass and peripheral indurated tissue provided adequate specimens for diagnosis. Communication with pathology about clinical suspicion for syphilis is essential, as spirochetes are not visible on routine hematoxylin and eosin staining. Frozen section analysis is therefore not adequate for the diagnosis of syphilis. Permanent pathology should be obtained prior to partial penectomy to ensure accurate diagnosis and potentially avoid organ loss, given that gummas may respond to treatment. While partial penectomy may ultimately be recommended, biopsy can help guide treatment and inform patient decisions. In the case of our patient, he was unwilling to proceed with surgery in the setting of a non-cancerous diagnosis.

## 4 Conclusion

With the resurgence of syphilis, tertiary syphilis should remain in the differential diagnosis of a penile mass, and penile mass biopsy can aid in diagnosis. This unique case highlights the strength of reverse algorithm screening and demonstrates a gummatous lesion so extreme that it did not respond to antibiotic treatment.

## Author disclosure statements

The following authors have nothing to disclose: GEM, SW, NSM, DX, HR, MRH, BRH.

## CRediT authorship contribution statement

**Gabriel E. Martin:** Writing – review & editing, Writing – original draft, Visualization, Project administration, Investigation, Data curation. **Sharon Wang:** Writing – review & editing, Conceptualization. **Nolan S. Maloney:** Investigation. **Dan Xu:** Investigation. **Herbert Ruckle:** Investigation. **Martin R. Hofmann:** Writing – review & editing, Investigation. **Brian R. Hu:** Writing – review & editing, Supervision, Conceptualization.

## Funding statement

This project received no funding.

## Declaration of competing interest

The authors declare that they have no known competing financial interests or personal relationships that could have appeared to influence the work reported in this paper.

## GLOSSARY

RPR: rapid plasma reagin
IHC: immunohistochemistry
DFA: direct fluorescent antibody
DFM: darkfield microscopy
EIA: enzyme immunoassay
CIA: chemiluminescence immunoassays
TP-PA: Treponema pallidum particle agglutination
FTA-ABS: fluorescent treponemal antibody absorption
VDRL: venereal disease research laboratory (test)
mL: milliliters
WBC: white blood cells
μL: microliter
dL: deciliter
CT: computed tomography
CFU: colony forming units
CDC: Centers for Disease Control and Prevention
